# Chlorovirus ATCV-1 Accelerates Motor Deterioration in SOD1-G93A Transgenic Mice and Its SOD1 Augments Induction of Inflammatory Factors From Murine Macrophages

**DOI:** 10.3389/fneur.2022.821166

**Published:** 2022-02-24

**Authors:** Thomas M. Petro, Irina V. Agarkova, Ahmed Esmael, David D. Dunigan, James L. Van Etten, Gary L. Pattee

**Affiliations:** ^1^Department of Oral Biology, University of Nebraska Medical Center, Lincoln, NE, United States; ^2^Nebraska Center for Virology, University of Nebraska Lincoln, Lincoln, NE, United States; ^3^Department Plant Pathology, University of Nebraska Lincoln, Lincoln, NE, United States; ^4^Botany and Microbiology Department, Faculty of Science, Benha University, Benha, Egypt; ^5^Neurology Associates P.C, Lincoln, NE, United States

**Keywords:** ALS, chlorovirus, ATCV-1, motor neuron diseases, SOD1-G93A mice

## Abstract

**Background:**

Genetically polymorphic Superoxide Dismutase 1 G93A (SOD1-G93A) underlies one form of familial Amyotrophic Lateral Sclerosis (ALS). Exposures from viruses may also contribute to ALS, possibly by stimulating immune factors, such as IL-6, Interferon Stimulated Genes, and Nitric Oxide. Recently, chlorovirus ATCV-1, which encodes a SOD1, was shown to replicate in macrophages and induce inflammatory factors.

**Objective:**

This study aimed to determine if ATCV-1 influences development of motor degeneration in an ALS mouse model and to assess whether SOD1 of ATCV-1 influences production of inflammatory factors from macrophages.

**Methods:**

Sera from sporadic ALS patients were screened for antibody to ATCV-1. Active or inactivated ATCV-1, saline, or a viral mimetic, polyinosinic:polycytidylic acid (poly I:C) were injected intracranially into transgenic mice expressing human SOD1-G93A- or C57Bl/6 mice. RAW264.7 mouse macrophage cells were transfected with a plasmid vector expressing ATCV-1 SOD1 or an empty vector prior to stimulation with poly I:C with or without Interferon-gamma (IFN-γ).

**Results:**

Serum from sporadic ALS patients had significantly more IgG1 antibody directed against ATCV-1 than healthy controls. Infection of SOD1-G93A mice with active ATCV-1 significantly accelerated onset of motor loss, as measured by tail paralysis, hind limb tucking, righting reflex, and latency to fall in a hanging cage-lid test, but did not significantly affect mortality when compared to saline-treated transgenics. By contrast, poly I:C treatment significantly lengthened survival time but only minimally slowed onset of motor loss, while heat-inactivated ATCV-1 did not affect motor loss or survival. ATCV-1 SOD1 significantly increased expression of IL-6, IL-10, ISG promoter activity, and production of Nitric Oxide from RAW264.7 cells.

**Conclusion:**

ATCV-1 chlorovirus encoding an endogenous SOD1 accelerates pathogenesis but not mortality, while poly I:C that stimulates antiviral immune responses delays mortality in an ALS mouse model. ATCV-1 SOD1 enhances induction of inflammatory factors from macrophages.

## Introduction

Infectious agents, such as viruses, have been postulated as contributors to motor neuron diseases (MND) (1). Documented infectious agents associated with MND include, West Nile virus, poliovirus, human immunodeficiency virus, and toxin-producing, cyanobacteria (2). Chloroviruses (CVs) represent an environmental agent recently associated with certain human pathogenic conditions (3–5). CVs are large dsDNA viruses, located ubiquitously within fresh-water environments worldwide, that infect and replicate in specific chlorella-like green algae (6). CV virions have been shown to infect mammalian macrophages *in vitro*, inducing inflammatory cytokines (7), and upon infection of mice induce neurocognitive deficits (3, 5). *Acanthocystis turfacea* chlorella virus 1 (ATCV-1; genus *Chlorovirus*, family *Phycodnaviridae*), is a member of one of four known CV types, and interestingly encodes a functional Cu-Zn superoxide dismutase (SOD) type 1 (8). ATCV-1 SOD1 is predicted to lower the concentration of reactive oxygen species in cells early during viral infection, thereby evading that aspect of innate anti-viral resistance (9). This is of clinical interest given that one of the most common genetic forms of amyotrophic lateral sclerosis (ALS) is due to polymorphisms in the human SOD1 gene, with mouse models recapitulating these findings (10).

SOD1-G93A transgenic mice are the most extensively studied ALS animal model. These transgenic mice express the human SOD1 disease variant containing an alanine for glycine mutation at amino acid 93 leading to symptoms of MND beginning at 150 days and resulting in 100% mortality by approximately day 170 (10–12). Similar to human ALS, development of MND in these mice occurs simultaneously with heightened expression of immune factors, such as IL-6 (13), Interferon Stimulated Gene (ISG) proteins (14), and Nitric Oxide (15), all of which have been shown to play a role in MND/ALS. The importance of abnormal SOD1 in ALS is also noted in sporadic ALS, which accounts for most ALS cases and where some degree of SOD1 dysfunction has been found (16). It is therefore intriguing to investigate the influence of ATCV-1 SOD1 in ALS.

The hypothesized pathogenic effect of ATCV-1 on humans is a relatively recent discovery (3). ATCV-1 was thought to only infect certain eukaryotic chlorella-like green algae, which are endosymbionts of the heliozoan *Acanthocystis turfacea*, a common protist of inland waters throughout the world (6). Infectious CVs found in inland aqueous environments are at times quite rare, but at other times show major spikes in abundance (up to 10^5^ infectious particles per ml of indigenous water). Forty-three CV isolates have been sequenced to date and classified into 4 basic groups (17, 18). However, not all aquatic systems contain CVs from each of these groups. Nevertheless, the chance of human exposure at some point to any one of the CVs should be quite high. It now appears that exposure to CVs is indeed significant following the detection of CV DNA sequences in the human virome (19, 20).

Since finding ATCV-1-like DNA sequences in oropharyngeal swabs from 43% of apparently healthy humans (3), seropositivity to ATCV-1 proteins was also found, suggesting immunologically significant exposures to this virus in humans (5). We further discovered an unexpected connection between ATCV-1, inflammatory immune responses, and cognitive impairments in otherwise healthy mice and humans. The presence of ATCV-1 DNA was not associated with specific demographic variables in this apparently healthy human cohort but was associated with statistically significant decreases in performance on cognitive assessments of visual processing and visual-motor speed. Additionally, mice orally inoculated with ATCV-1-infected algae exhibit immune responses to viral proteins, significant changes in transcriptional patterns of endogenous genes within the hippocampus, and similar cognitive impairments (3). Infectious ATCV-1, as measured by plaque assay, was recovered from spleens of C57Bl/6 mice 4 weeks after the original oral gavage inoculation (Yolken et al., personal communication). We have subsequently reported that mouse macrophages support low levels of ATCV-1 replication *in vitro*, which was accompanied by the production of inflammatory cytokines (7). This led us to hypothesize that ATCV-1 persistence in macrophages inducing inflammatory cytokine production is a mechanism by which CVs could impair neurological function. This hypothesis is a corollary of the well-established theory that high levels of neuroinflammation impair cognition, behavior, and neurological function (21–23). It is therefore plausible that persistence of ATCV-1 in macrophages stimulates the production of immune factors contributing to neurodegenerative disease (24, 25), such as Nitric Oxide (NO), IL-6 (26), or ISGs (14). In this report, we evaluate the hypothesis that exposure to and/or infection with ATCV-1 may lead to the development of motor neuron diseases, such as ALS. We show that a cohort of ALS patients had higher than normal IgG1 antibody to ATCV-1, degradation of motor performance is accelerated in ATCV-1 challenged SOD1-G93A transgenic mice. Moreover, expression of ATCV-1 SOD1 in responding mouse macrophages resulted in enhanced NO, IL-6, and IL-10 protein production and ISG promoter activity. Surprisingly, poly I:C a powerful stimulant of innate anti-viral immune responses and is used here as a control, significantly delayed mortality in SOD1-G93A transgenic mice.

## Methods and Materials

### Human Serology

Following enrollment in a clinical ALS research study and after IRB approval (IRB #: 20160515832EP), serum samples were collected utilizing standard blood drawing procedures from 17 patients with sporadic ALS and 13 age matched controls, voluntarily and randomly collected from the local population and ALS clinics. These samples underwent evaluation for IgG1, IgG2, IgG3, IgG4, IgA, and IgM antibody isotypes/subclasses directed against ATCV-1 using ELISA techniques. Briefly, 96-well ELISA plates were coated with 2 μg/ml ATCV-1 protein or phosphate buffered saline (PBS). After overnight incubation at 4°C, plates were washed and blocked with Superblock (Thermo-Fisher #3715 and 3716). After blocking, individual sera from ALS patients and healthy controls were diluted in Superblock from 1:100 to 1:1000 and added to ATCV-1 or PBS wells. After 2 h incubation at room temperature, wells were washed and then incubated with peroxidase-labeled anti-human IgG1, IgG2, IgG3, IgG4, IgA, or IgM (Southern Biotech, Birmingham, Alabama), after which wells were washed and then TMB reagent was added. To determine relative antibody levels, OD450 nm of the 1:500 diluted sera was measured using an ELISA plate reader and absorbance in wells with PBS was subtracted from wells coated with ATCV-1.

### Mouse Model Experiment

Female SOD1-G93A-transgenic mice and C57Bl/6 wild-type mice at 5 weeks of age were purchased from Jackson Labs, Bar Harbor, ME and randomly assigned to experimental groups. Mice were injected intracranially (i.c.) at 5 weeks of age with 50 μl of phosphate-buffered saline (PBS) containing 5 x 10^8^ ultra-purified ATCV-1 plaque-forming virus particles as assayed on the alga *Chlorella heliozoae* SAG 3.83, 50 μg of poly I:C to induce innate antiviral immune responses, or no additional components, as previously described (4). Briefly, following isoflurane anesthetization, ATCV-1 in PBS in a volume of 50 μl was injected with a 27 gauge $\frac{1}{4}$ inch needle 3 mm below the surface of the skull ~1.0 mm anterior to the bregma and 1.0 mm lateral to the sagittal suture. The injection was performed over a period of 2. Following injection, topical Bupivacaine was administered periodically at the injection site for the first 24 h. These transgenic mice express human SOD1-G93A and develop ALS-like MND morbidity and mortality within 150–170 days from birth. The mice were monitored daily for initiation and propagation of motor dysfunction and subsequent mortality. Motor dysfunction analysis included detailed daily physical examinations of the animals, assessing the time of onset of tail paralysis, hindlimb tucking and decrease in righting reflex. These clinical responses were measured and recorded using an established four-point animal motor analysis scale: level (1) Collapse/partial collapse of the hind-legs toward midline; (2) Toe curl during walking or foot-dragging; (3) Rigid hind limb paralysis or minimal joint movement for forward motion; (4) Failure to right within 30 s for all animals (27). Starting at 90 days, separate cohorts of mice were given the hanging cage lid test of motor neuron function. Briefly, individual mice were placed onto the grid of a standard mouse cage lid and after the lid was inverted above an empty padded cage, the time at which the mice dropped to the cage floor was recorded as the latency to fall, for a maximum of 180 s.

### Cells and Transfection

RAW264.7 Lucia cells were purchased from Invivogen and maintained in Dulbecco's Modified Eagle Medium (DMEM)-high glucose culture media (Invitrogen) containing 10% fetal bovine serum and 50 μg/ml gentamicin. RAW Lucia cells were harvested from cell cultures, quantified using a hemacytometer and trypan blue staining, adjusted to 1 x 10^5^ cells/ 500 μl per well of a 24-well cell culture plate in complete culture media, and incubated overnight at 37°C in 5% CO_2_. Using Lipofectamine 3000 (Invitrogen), cells were then transfected, according to manufacturer's specification, with 0.5 μg of plasmid empty vector (pGL3) or plasmid expression vector (pCMV6-entry) containing the inserted coding sequence of ATCV-1 SOD1 (Z190L) (8) purchased from Blue Heron (Bothell, WA, USA). Cells undergoing transfection were re-incubated for 24 h prior to stimulation with 10 μg/ml of poly I:C (InVivogen, San Diego, CA, USA) with or without 40 pg/ml of mouse IFN-γ (Cell Signaling Technology, Danvers, MA, USA). After an additional 18 h of incubation at 37°C/5% CO_2_, secreted luciferase in 10 μl of supernatant was measured using the QuantiLuc kit (InVivogen). After an additional 6 h, supernatants were collected and used for IL-6 and IL-10 ELISA and Nitric Oxide Griess assays.

### IL-6 and IL-10 ELISA

The Mouse IL-6 and IL-10 ELISA kits (Invitrogen/ThermoFisher, Waltham, MA, USA) were used to determine the concentration of cytokine in RAW264.7 Lucia culture supernatants according to manufacturer's specifications. Briefly, capture antibody was diluted 1:250 in binding buffer and 100 μl was applied to 96-well ELISA plates overnight at 4°C. After washing with PBS/0.05% Tween 20, wells were blocked with ELISA/ELISPOT diluent for 1 h. After washing, 100 μl of individual supernatant solutions or 100 μl of recombinant standard IL-6 or IL-10 was applied to individual wells and incubated at room temperature for 2 h, washed 3X, after which a 1:250 dilution of biotin-labeled detection anti-IL-6 was applied to each well and incubated for 1 h. Following 3X washing, diluted avidin peroxidase was added to each well and incubated for 30 min. Substrate tetramethylbenzidine (TMB) was added to each well after 5X washes and incubated for 15 min, after which 50 μl of stop solution was applied and then optical densities at 450nm were determined using an ELISA plate reader. Concentrations of IL-6 in supernatants were determined by using a regression analysis of the values from the standards.

### Griess Assay of Nitric Oxide

Using a Griess assay kit from Invitrogen/ThermoFisher, 150 μl of supernatant or standards were mixed with 20 μl of Griess reagent for 30 min at RT. Optical densities at 550 nm were determined using an ELISA plate reader. Concentrations of Nitric Oxide in the supernatants were determined by using a regression analysis of the values from the standards.

### Statistical Analysis

Data are reported as means ± standard errors. The significance of differences between means were determined using the Student's *t*-test, with *p* ≤ 0.05 considered to be significant.

## Results

### Anti-ATCV-1 IgG1 and Inflammatory Cytokines in Sporadic ALS Patients

**Figure 1:**
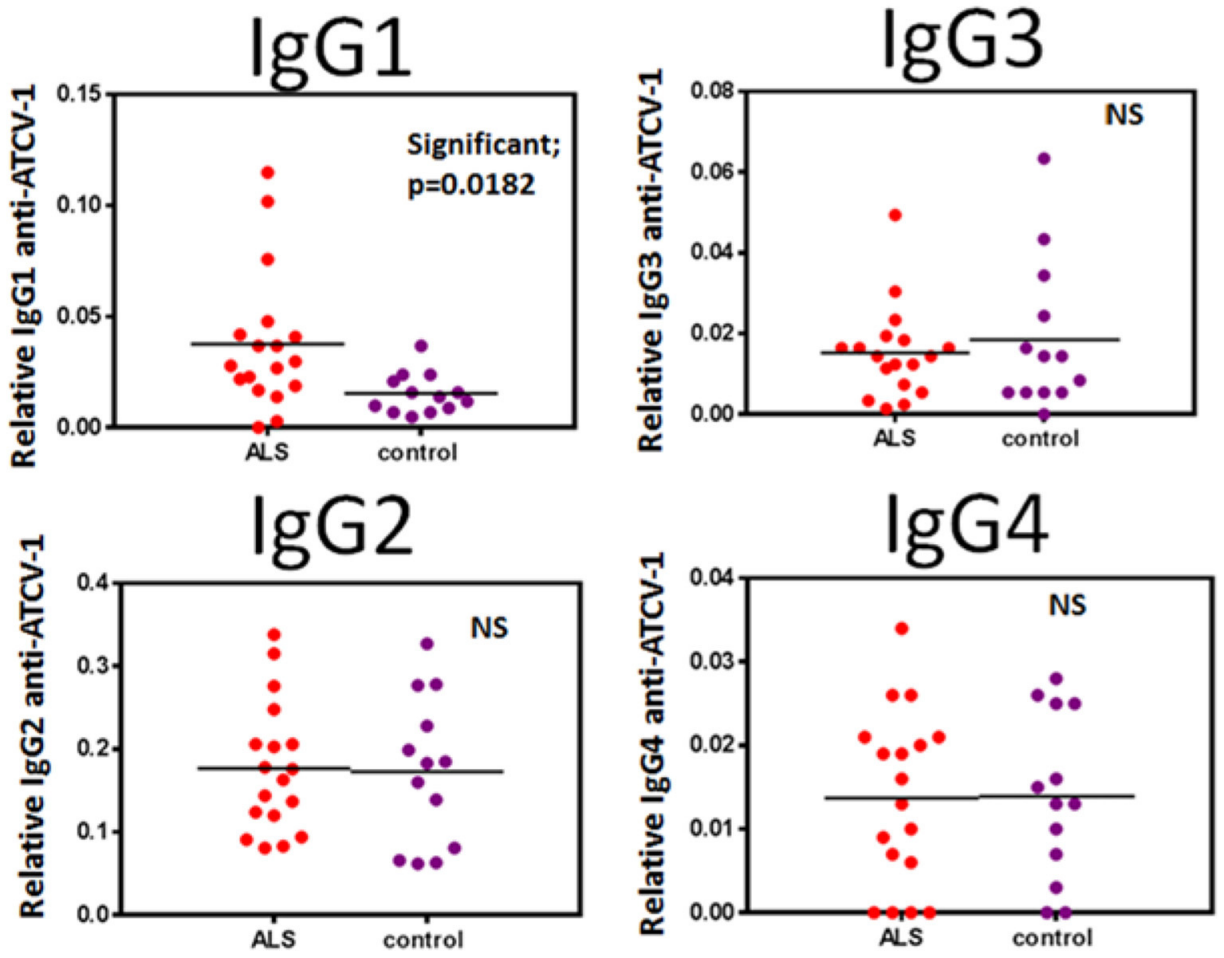
Sera from ALS patients (*n* = 18) and healthy control subjects (*n* = 13) were analyzed by IgG subclass (IgG1, IgG2, IgG3, IgG4) for reactivity to ATCV-1 virus-coated ELISA plates. Data were analyzed for statistical significance using Student's *t*-test with the GraphPad Prism software. Significance is *p* < 0.05.

Significant exposures to viruses are detected by measuring antibody that is reactive to the specific virion, most often done using ELISA protocols. We therefore measured human anti-ATCV-1 IgG subclasses in serum from ALS patients as compared to healthy controls. Anti-ATCV-1 IgG1, but not other subclasses of IgG, was significantly higher in serum of ALS patients compared with controls (Figure 1). ALS patients would therefore appear to have had a significantly greater exposure to ATCV-1 or a greater antibody response to a similar level of exposure, compared with healthy controls.

### Chlorovirus ATCV-1 and MND in SOD1G93A Transgenic Mice

**Figure 2:**
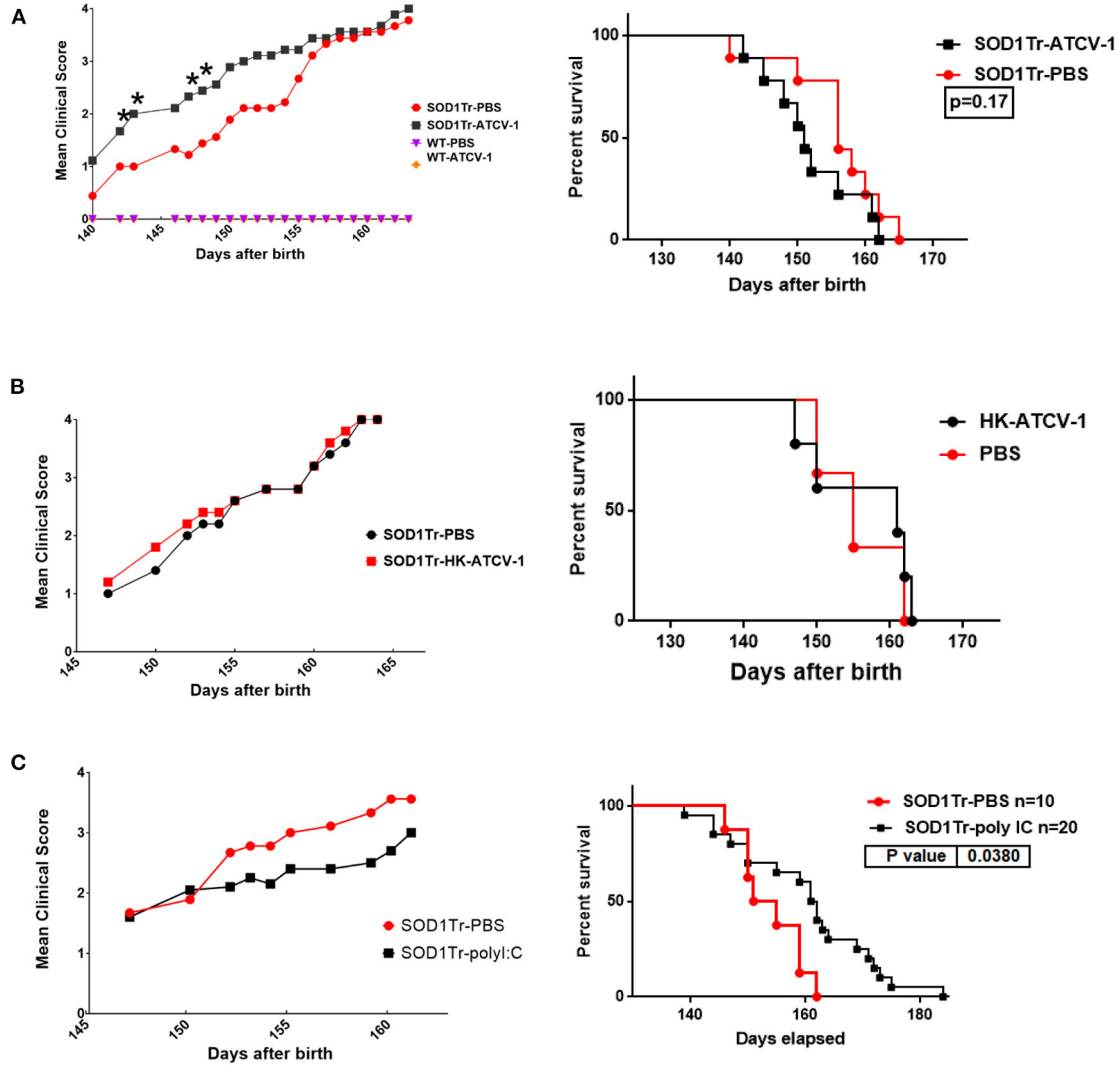
**(A)** SOD1-G93A transgenic mice and control wild-type (wt) C57Bl/6 mice were inoculated i.c. with 5 x 10^8^ plaque forming unit viral particles of ATCV-1 (*n* = 9; 50 μl) or PBS (*n* = 9; 50 μl) at 35 days of age. **(B)** A second cohort of SOD1-G93A transgenic mice were inoculated i.c with PBS (*n* = 5) or 5 x 10^8^ heat-killed (HK) ATCV-1 (*n* = 5). **(C)** A third cohort of SOD1G93A transgenic mice were inoculated i.c with PBS (*n* = 10) or poly I:C(20 ug) (*n* = 20). (Left graphs) MND evaluation: level 1: Collapse/partial collapse of hind leg toward midline; level 2: Toe curl during walking or foot-dragging; level 3: Rigid hind limb paralysis or minimal joint movement for forward motion; level 4: Failure to right within 30 s. (data analyzed by Student's *t*-test; * indicates significance *p* ≤ 0.05), (Right graphs) Kaplan-Meier Survival data of transgenic inoculated mice.

In a preliminary experiment we orally gavaged mice with ATCV-1 and then determined at 8 days post infection plaque forming units (pfu) of ATCV-1 in spleen, liver, brain, and intestinal homogenates. We found ATCV-1 pfu in spleen, liver and brain homogenates of these mice (data not shown), suggesting that ATCV-1 is capable of transiting the blood-brain barrier. To determine the impact of ATCV-1 on MND we used a mouse model in which SOD1-G93A-transgenic mice develop ALS-like MND around 150 days of age and progress to severe morbidity and mortality by day 170. SOD1-G93A-transgenic mice or C57Bl/6 control mice were i.c. injected at day 35 of age with ATCV-1, PBS, poly I:C, or heat-killed (HK) ATCV-1. Poly I:C was used as a control since it induces innate antiviral immune responses without viral infection. The results reveal that ATCV-1 infection significantly accelerated onset and progression of MND in SOD1-G93A-transgenic mice starting at day 141, compared to the PBS-treated transgenic mice, which developed symptoms of MND starting at 150 days (Figure 2A). Significantly, greater motor deterioration of ATCV-1-infected mice was detected for the next 14 days, after which the MND in ATCV-1-inoculated SOD1G93A-transgenic mice approached that of the PBS-treated transgenic mice. In contrast to MND, accelerated mortality was not noted for ATCV-1-infected SOD1-G93A-transgenic mice compared with PBS-treated transgenics (Figure 2A). Also, HK-ATCV-1 did not affect motor deterioration or mortality in SOD1-G93A-transgenic mice compared with PBS-treated transgenic mice (Figure 2B). Surprisingly, treatment with poly I:C resulted in a slight improvement in motor deterioration and a significant delay in mortality in SOD1-G93A-transgenic mice compared with transgenic mice receiving PBS (Figure 2C). C57Bl/6 (WT) mice inoculated with ATCV-1 or injected with PBS did not exhibit any loss of motor function or any mortality during the course of these experiments.

**Figure 3:**
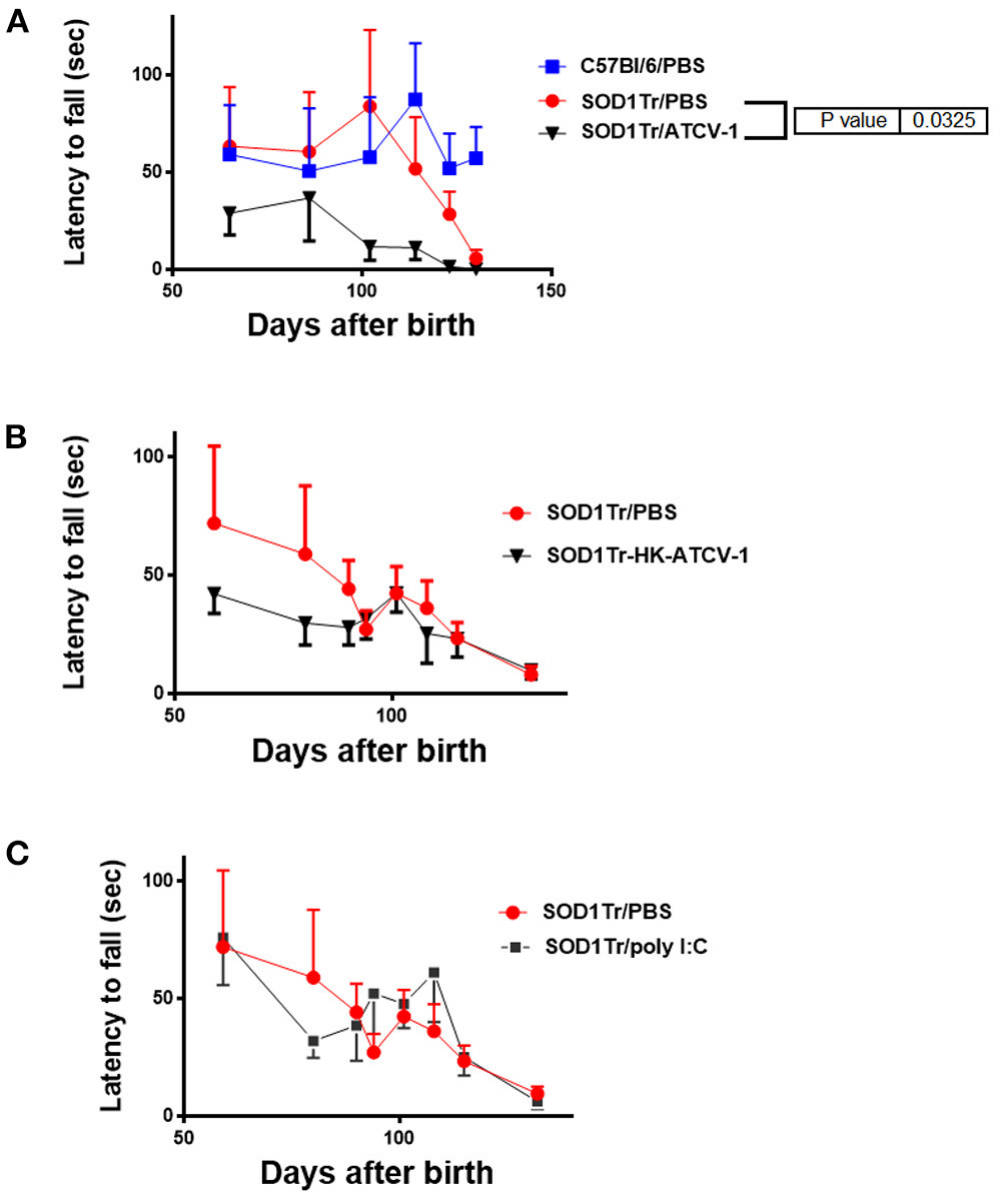
**(A)** SOD1-G93A-transgenic mice and control wild-type (wt) C57Bl/6 mice were inoculated i.c. with 5 x 10^8^ PFU of ATCV-1 (*n* = 9; 50 μl) or PBS (*n* = 9; 50 μl) at 35 days of age. **(B)** A second cohort of SOD1G93A transgenic mice were inoculated i.c with PBS (*n* = 5) or 5 x 10^8^ heat-killed (HK) ATCV-1 (*n* = 5). **(C)** A third cohort of SOD1G93A transgenic mice were inoculated i.c with PBS (*n* = 10) or poly I:C(20 μg) (*n* = 20). Latency (sec) to fall of inoculated transgenic or wt mice after hanging from an cage lid (data analyzed by 2-way ANOVA using GraphPad Prism software).

To confirm these findings, separate groups of ATCV-1, poly I:C, HK-ATCV-1, and PBS inoculated mice were evaluated using the hanging cage lid latency to fall test, which detects MND in SOD1-G93A transgenic mice at earlier stages (28). Using this technique, MND as measured by latency to fall in PBS-treated SOD1-G93A-transgenic mice was detected as early as day 114. Significant decreases in latency to fall in ATCV-1-inoculated transgenic mice was detected at around day 100 and overall ATCV-1-inoculated transgenic mice exhibited a significant decline in latency compared with PBS treated SOD1-G93A transgenic mice, as measured by ANOVA (Figure 3A). Transgenic mice receiving HK-ATCV-1 (Figure 3B) or poly I:C (Figure 3C) did not exhibit significant changes in latency to fall, as compared with PBS-treated SOD1-G93A transgenics. These data demonstrate that the hanging cage-lid latency to fall test is a more sensitive measure of MND in mice and confirm that ATCV-1 infection accelerates motor deterioration in SOD1-G93A transgenic mice.

### ATCV-1 SOD1 Enhances Macrophage Expression of Immune Factors

**Figure 4:**
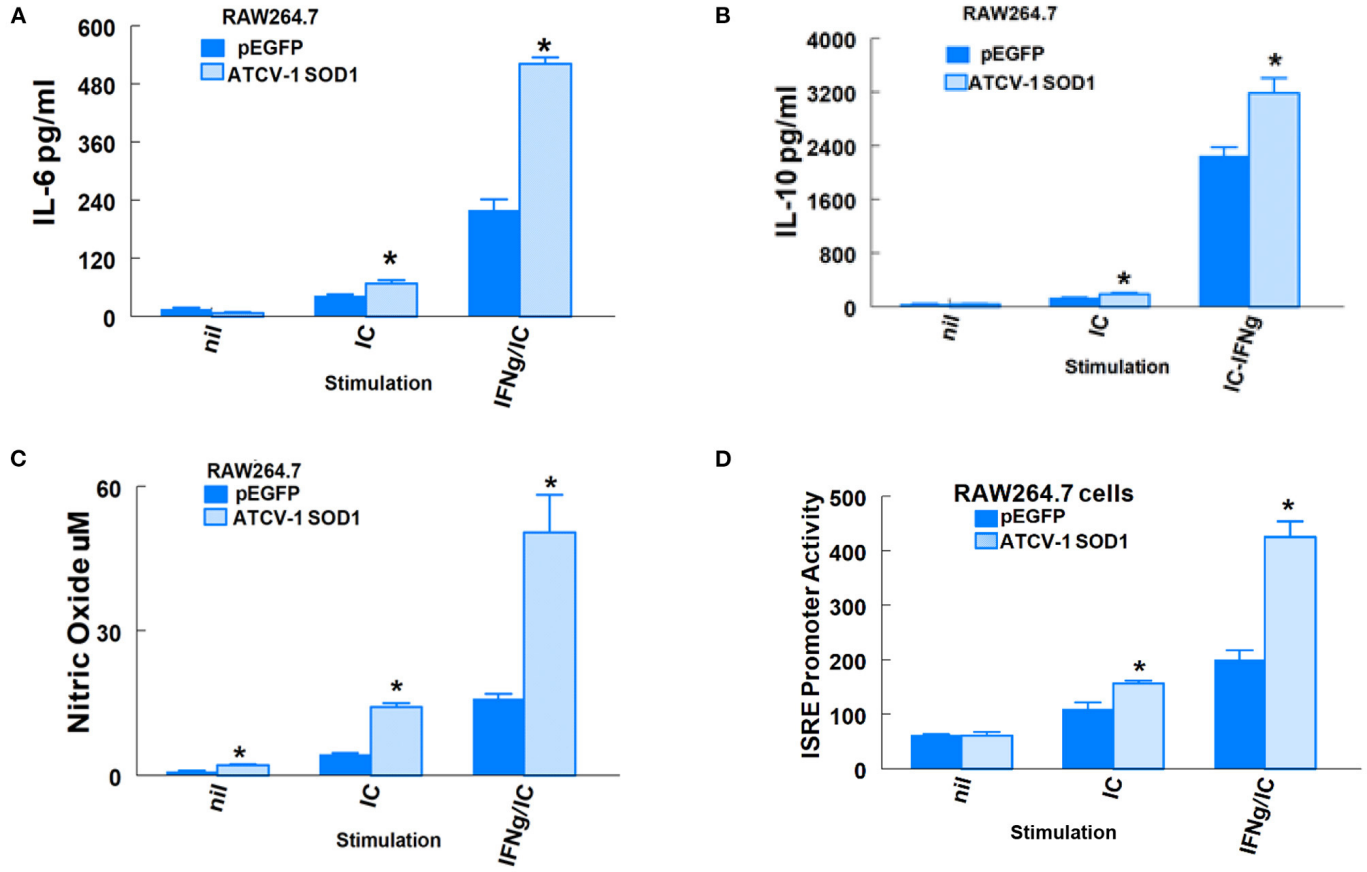
ATCV-1 SOD1 and expression of immune factors from RAW264.7 Lucia cells. **(A)** IL-6, **(B)** IL-10, **(C)** Nitric Oxide (NO), and **(D)** Secreted Luciferase driven by an ISRE promoter in supernatants of RAW264.7 cells transfected with pEGFP empty vector or an expression vector coding for ATCV-1 SOD1 and then stimulated with 10 ug/ml poly I:C (IC) with or without 50 ng/ml mouse recombinant IFN-gamma (IFN-γ). Representative data from three experiments with means ± standard error; *n* = 4 separate transfections for each experiment. * indicates significantly different from cells transfected with empty vector.

In addition to SOD1-G93A, immune system factors such as IL-6, ISGs, and NO (29) are associated with ALS in humans and MND in SOD1-G93A transgenic mice. We showed previously that i.c. inoculation of mice with ATCV-1 led to persistence of viral nucleic acid in the brain and higher than normal inflammatory factors, such as IFN-gamma, and macrophage microglial markers, such as CD11b for up to 8 weeks (4). Moreover, ATCV-1 encodes its own SOD1 (8). Since we previously showed that ATCV-1 infects macrophages and induces IL-6 and NO (7), we set out to determine if the SOD1 of ATCV-1 could influence IL-6, IL-10, and NO expression, as well as ISG promoter activity in macrophages. We used the RAW264.7 Lucia mouse macrophage cell line that constitutively contains a plasmid with a promoter-reporter system expressing secreted Luciferase under the control of ISRE elements (5X). ISRE promoter elements are found in promoters of ISG genes like ISG54 (30) or ISG15 (31). RAW264.7 Lucia cells were first transfected with a plasmid vector that expresses ATCV-1 SOD1 or an empty vector. Following transfection, cells were unstimulated or stimulated with poly I:C in the presence or absence of IFN-γ to induce production of IL-6, IL-10, and NO with activation of the ISG promoter reporter. The results clearly show that expression of ATCV-1 SOD1 significantly enhanced activation of the ISG promoter in response to poly I:C with or without IFN-γ compared with RAW Lucia cells that were transfected with an empty vector (Figure 4D). Similarly, production of IL-6, IL-10, and NO were significantly higher in RAW Lucia cells transfected with ATCV-1 SOD1 expression vector compared with empty vector (Figures 4A–C). In summary, these results indicate that ATCV-1 SOD1 augments production of inflammatory factors IL-6 and NO, and ISG expression, which are associated with development of ALS in humans and MND in SODG93A transgenic mice.

## Discussion

The data here do not support our original hypothesis presented that exposure to and/or infection with ATCV-1 may lead to the development of MND, such as ALS. However, the data here support a revised hypothesis that ATCV-1 intracranial exposure in SOD1 transgenic mice accelerates disease onset and progression of MND, but not mortality. To establish the possible pathogenesis of CVs, we performed ALS mouse model studies, which identified a significant impact of ATCV-1 on MND onset and progression in SOD1-G93A transgenic mice. Our results indicate that only active ATCV-1 compared with inactivated ATCV-1 hastened the onset of MND in SOD1-G93A mice. Nevertheless, while ATCV-1 infection increased the severity of MND it did not alter survival significantly. Given that treatment with poly I:C delayed onset of MND, it is plausible that active ATCV-1 infection or some component of the anti-viral immune response against active viral infection is promoting early onset of MND. Our previous studies showed that mouse macrophage challenged with ATCV-1 respond with increased production of innate antiviral immune factors such as IL-6 and NO, in addition to viral proteins and active virions. A subsequent investigation showed that 8 weeks after i.c. inoculation of ATCV-1 in C57Bl/6 mice detectable ATCV-1 gene expression as well as heightened inflammatory markers, such as iNOS, Interferon-gamma, and microglial marker CD11b, were found in the brain (4). These findings led us to research the possible triggers for ATCV-1 induction of immune factors. In our prior studies (7) we detected two putative ATCV-1 proteins whose production was dominant during infection of macrophages. A 55kD protein, which corresponds to the size of ATCV-1 major capsid protein and a 17kD protein, which corresponds to the size of ATCV-1 SOD1. Herein we show in the RAW Lucia macrophage cell line that overexpression of ATCV-1 SOD1 significantly enhances innate immune factors, such as IL-6, IL-10, NO, and ISG promoter activity. Therefore, it remains likely that ATCV-1 contributes to the acceleration of MND in SOD1-G93A transgenic mice, at least in part through its SOD1 enhancement of immune factors. This is highly significant because heightened IL-6, NO, and ISG15 have been associated with the development of ALS in humans and MND in SOD1-G93A transgenic mice (13–15). However, it is not unexpected that overexpression of SOD1 would trigger MND. Moreover, Alexander et al. (32), showed that copy number of SOD1 G93A was correlated with the degree of disease severity in transgenic mice, and even high expression levels of non-mutated SOD1 can contribute to MND in mice (33).

Of particular interest is Interferon-stimulated-gene 15 (ISG15), as previous studies have shown that ISG15 production is progressively enhanced in the spinal cord of SOD1-G93A mice starting at pre-symptomatic day 60 (14). In this same study, spinal cords of ALS patients exhibited elevated ISG15 protein levels compared with spinal cords of controls. ISG15 is one of dozens of ISGs that are induced during the response of cells to virus infection and innate anti-viral immune responses likely trigger ALS (34). While most ISGs are directly anti-viral, ISG15 is an ubiquitin-like protein that ISGylates several other proteins involved in anti-viral immune responses, including IRF3 (35). In this case, ISGylation of IRF3 protects it from ubiquitination and proteosomal degradation, thereby critically sustaining IRF3 activity during viral infection (36). Activated IRF3 for its part is pivotal to the expression of key innate antiviral immune components, such as IFN-beta (37), IL-6 (38), NO (39), and ISG15 itself. While these antiviral factors are needed to control virus infection they are also involved in the pathology of ALS. Thus, the SOD1 of ATCV-1 enhances production of anti-viral immune factors that may contribute to the development of ALS.

The mechanisms by which humans come to be exposed to chloroviruses like ATCV-1 remain largely unknown. However, respiratory inoculation or oral ingestion may prove to be a significant means of exposure. Our preliminary results show that oral gavage of ATCV-1 in mice results in detectable ATCV-1 in brain homogenates 8 days post infection (data not shown). Given that measurable serum antibody titers against ATCV-1 in adult humans have been detected (data not shown), a common means of exposure throughout the general population would evidently exist and some of the inoculum may transit through the blood-brain barrier.

The limitations of this study include the fact that only one ALS animal transgenic model was studied and other transgenic mice with polymorphic human genes, such as C9orf72 (40) or SOD1-G37R (41), should also be assessed. In addition, the contribution of other types of CVs on transgenic animal models and other routes of inoculation, such as intranasal, intraoral, and subcutaneous should also be assessed. The timing of the CV inoculations either early or later in the transgenic life cycle, prior to the disease onset should also be studied. Direct ATCV-1 infections of neuronal cell lines in the context of SOD1-G93A overexpression are currently under investigation to better understand if and how these viruses contribute to MND through a specific cell type.

Our findings raise several important clinical questions necessitating clarification from future studies. It is imperative to better understand exactly how humans are routinely exposed to CVs and to what extent elevated CV antibody titers could be found in ALS patients when compared with healthy subjects. Moreover, it is important to assess to what degree do CVs cross the blood-brain barrier and does exposure to these viruses early in life or incorporation of CVs within the gut microbiome with subsequent replication, pose a risk factor for, or protection from, disease development later in life. It is also essential to determine the roles and involvement of macrophages in virus-induced MND.

In conclusion, these study data identify that exposure to ATCV-1 may accelerate the onset of an ALS-like MND in a transgenic mouse model and its SOD1 augments induction of inflammatory factors from macrophages. Our cumulative research findings to date would indicate that SOD1 encoding CVs, ubiquitous worldwide in freshwater environments, may contribute to the pathogenesis of ALS. Ongoing investigative efforts are now underway to better understand and define this apparent association.

## Data Availability Statement

The raw data supporting the conclusions of this article will be made available by the authors, without undue reservation.

## Ethics Statement

The animal study was reviewed and approved by IACUC of the University of Nebraska Medical Center.

## Author Contributions

TP contributed to the conception, design, project implementation, and manuscript preparation. JV and DD contributed to the conception, design, and manuscript revision. IA contributed to the conception, virus preparation, and manuscript revision. AE contributed to interpretation of the data, experiment validation, and manuscript revision. GP contributed to conception, manuscript preparation, and revision. All authors contributed to the article and approved the submitted version.

## Funding

This study was funded by Stuart Nichols Research Foundation, the University of Nebraska-Lincoln Agricultural Research Division and the Office of Research and Economic Development for DD's salary.

## Conflict of Interest

The authors declare that the research was conducted in the absence of any commercial or financial relationships that could be construed as a potential conflict of interest.

## Publisher's Note

All claims expressed in this article are solely those of the authors and do not necessarily represent those of their affiliated organizations, or those of the publisher, the editors and the reviewers. Any product that may be evaluated in this article, or claim that may be made by its manufacturer, is not guaranteed or endorsed by the publisher.

## Abbreviations

ALS: Amyotrophic Lateral Sclerosis
SOD1: superoxide dismutase 1
IFN: Interferon
IL: Interleukin
ISG: IFN-stimulated gene
NO: nitric oxide
IFN-γ: IFN-gamma
MND: Motor Neuron Disease
CV: chlorovirus
ATCV-1: Acanthocystis turfacea chlorella virus 1
HK: heat-killed
PBS: phosphate-buffered saline.
